# Altered Theory of Mind Engagement and Neural Alignment in Social Anxiety During Movie Viewing

**DOI:** 10.1016/j.bpsgos.2026.100721

**Published:** 2026-03-14

**Authors:** Saskia B.J. Koch, Margot Mangnus, Robin Devillers, Peter Hagoort, Jana Bašnáková, Arjen Stolk

**Affiliations:** a Donders Institute for Brain, Cognition, and Behaviour, Radboud University Nijmegen, Nijmegen, the Netherlands; b Behavioral Science Institute, Radboud University Nijmegen, Nijmegen, the Netherlands; c Max Planck Institute for Psycholinguistics, Nijmegen, the Netherlands; d Institute of Experimental Psychology, Center of Social and Psychological Sciences, Slovak Academy of Sciences, Bratislava, Slovakia; e Psychological and Brain Sciences, Dartmouth College, Hanover, New Hampshire

**Keywords:** Functional magnetic resonance imaging, Interpretation bias, Intersubject correlation, Naturalistic neuroscience, Pupillometry, Social anxiety, Theory of mind

## Abstract

**Background:**

Social anxiety (SA) is marked by a persistent fear of social scrutiny, but its neurocognitive mechanisms remain incompletely understood. We tested whether SA is associated with altered engagement of theory of mind (ToM) systems supporting inferences about others’ thoughts and emotions or with broader interpretive tendencies biasing social information processing toward more internally guided responses.

**Methods:**

Functional magnetic resonance imaging, heart rate, and pupil diameter were recorded from 43 individuals with elevated SA and 43 control individuals with low SA during viewing of a nonverbal animated film. ToM engagement was assessed via scene-specific neural activation, while broader interpretive processing was indexed using dynamic intersubject correlation (ISC), quantifying the degree to which neural responses were shared versus idiosyncratic across viewers over time.

**Results:**

Participants with elevated SA showed reduced activation in the left posterior superior temporal sulcus during ToM-relevant scenes, as well as preserved engagement of the broader ToM network. Dynamic ISC analyses revealed increased alignment in early sensory regions and reduced alignment in higher-order regions outside the ToM network, consistent with more individualized processing at higher levels. These effects occurred without group differences in autonomic arousal or pupil-linked attention, and an exploratory comparison with an autism cohort revealed opposite alignment patterns in overlapping higher-order regions.

**Conclusions:**

SA is associated with focal alterations in ToM-related processing and broader shifts in movie-driven neural alignment across the cortical hierarchy. Divergent alignment patterns in SA and autism suggest that movie-driven neural alignment may serve as a transdiagnostic marker distinguishing mechanistically distinct forms of atypical social cognition.

## Plain Language Summary

Social anxiety is marked by fear of being judged by others, but it remains unclear how it shapes the interpretation of social situations. We measured brain activity, heart rate, and eye responses while people with higher or lower social anxiety watched a short animated film depicting social interactions. Individuals with elevated social anxiety showed subtle changes in brain activity related to understanding others, as well as more individualized brain responses during the movie, even without signs of increased arousal or attention. These findings suggest that social anxiety alters how social information is processed even in situations that are not overtly threatening.

Social anxiety (SA) disorder, or social phobia, is characterized by a persistent and excessive fear of being judged or scrutinized in social situations (1,2). Typically emerging in childhood or adolescence and affecting approximately 1 in 8 adults, the condition is associated with substantial impairment in daily functioning (3,4). However, beyond its threat-related features, the neurocognitive mechanisms shaping how social information is processed in SA remain incompletely understood (5). Here, we focus on two candidate sociocognitive processes implicated in the condition: theory of mind (ToM) and broader interpretive biases.

ToM, or mentalizing, refers to the capacity to infer others’ unobservable thoughts and emotions, supporting the interpretation and prediction of social behavior (6,7). In SA, ToM difficulties have been proposed to increase uncertainty about how one is perceived by others, thereby amplifying fears of negative evaluation (8, 9, 10). Consistent with this account, individuals with SA show reduced performance on ToM tasks such as the Reading the Mind in the Eyes Test and the Movie Assessment of Social Cognition (11, 12, 13, 14), as well as altered activity and connectivity in canonical ToM regions including the medial prefrontal cortex (mPFC), precuneus, temporoparietal junction (TPJ), and superior temporal sulcus (STS) (15, 16, 17, 18). However, other findings point to exaggerated interpretation of others’ emotional expressions and social signals (10,19,20), raising questions about whether ToM is underutilized, overengaged, or otherwise dysregulated in SA. Notably, much of this evidence derives from explicitly evaluative tasks that may themselves induce anxiety, leaving it unclear whether altered mentalizing reflects a core feature of the condition or context-dependent effects of evaluation.

A complementary body of work has focused on interpretive biases, defined as systematic tendencies to assign excessive personal significance to social cues (21, 22, 23, 24). Although models vary in emphasis, they converge on the idea that internally generated expectations of negative evaluation, often grounded in a negative self-image, disproportionately shape how external input is processed, both online and in retrospect (25, 26, 27, 28, 29, 30, 31, 32). Neuroimaging studies support this account, showing altered engagement of precuneus and frontoparietal regions during social feedback (33), as well as increased interindividual variability in neural responses during naturalistic movie viewing (34). Rather than reflecting noise or disengagement, such variability may index more individualized, internally guided processing of shared social input (35,36). However, it remains unclear to what extent these interpretive tendencies operate independently of threat-related arousal or attention (37) and how they relate to ToM computations supporting mental state inference, as few studies have examined these dimensions within a single experimental framework.

To address this gap, we used a movie-viewing paradigm that enables concurrent assessment of scene-specific mentalizing and broader interpretive tendencies under minimally evaluative conditions. Forty-three individuals with elevated SA and 43 control individuals with low SA viewed the animated short *Partly Cloudy* during functional magnetic resonance imaging (fMRI). The film depicts the development of a relationship between a stork and a cloud, conveyed entirely through nonverbal cues. Mental state events known to recruit the canonical ToM network (38, 39, 40) are embedded within a continuous stream of social information, enabling event-related analyses of ToM-relevant scenes alongside dynamic intersubject correlation (ISC) as an index of movie-driven neural alignment, reflecting the degree to which neural responses are shared versus individualized across viewers over time (41). Prior work using this paradigm in autism reported preserved ToM-related activation alongside elevated alignment in non-ToM regions across much of the film, suggesting a dissociation between scene-specific mentalizing and broader interpretive processing (42). Here, we applied this framework to examine how these processes manifest and interact in SA.

To further dissociate sociocognitive effects from general arousal or attention, we measured whole-brain activity together with heart rate and pupil diameter (43, 44, 45, 46) and collected an unannounced postviewing assessment of participants’ movie interpretations. Based on prior evidence for negativity bias in SA (32,47), we predicted greater use of negatively valenced emotion language in participants’ plot summaries but no a priori group differences in autonomic arousal or pupil-linked attentional measures, given the nonevaluative nature of the paradigm. At the neural level, we expected group differences in one or more core ToM regions during mentalizing events (12), as well as increased interindividual variability, expressed as reduced alignment, in frontoparietal regions previously implicated in SA (34). Finally, motivated by spatially overlapping ISC effects reported in autism using the same paradigm, we conducted a post hoc cross-study comparison to examine convergent and divergent patterns of neural alignment across the 2 conditions (48).

## Methods and Materials

### Participants

Eighty-six adults were recruited via Radboud University’s research database, social media platforms, and campus postings. Exclusion criteria included current use of psychotropic medication, severe cognitive impairment, systemic disease, or a history of neurological treatment. SA was assessed using the 24-item Liebowitz Social Anxiety Scale (LSAS) (49,50), which measures fear and avoidance across common social situations (e.g., eating in public, interacting with strangers) on a 4-point Likert scale. A validated cutoff score of 30 was used to classify participants into high-SA (LSAS ≥ 30) or low-SA (LSAS < 30) groups, yielding 43 individuals per group (50). Groups were matched on sex, age, and verbal and nonverbal IQ (51,52) (Table 1). Participants with low anxiety reported no current psychiatric diagnoses. MRI data were acquired from all participants (*N* = 86), heart rate recordings from 74 participants, pupillometry from 75 participants, and postviewing questionnaires from 84 participants. All participants provided verbal and written informed consent in accordance with local ethics guidelines and received monetary compensation for their participation. The study was approved by the local ethics committee (Committee for Human-Subject Research, region Arnhem-Nijmegen, the Netherlands; File No. 2019-6059).

**Table 1:** Demographic Characteristics

	Group		Group Difference		
	High SA, *n* = 43	Low SA, *n* = 43	Test Statistics		BF
Sex, Female	27 (62.79%)	25 (58.14%)	χ^2^_1_ = 0.05	*p* = .825	BF_01_ = 3.53
Age, Years	26.33 (5.92)	26.05 (5.28)	*t*_84_ = 0.23	*p* = .818	BF_01_ = 4.34
Verbal IQ, WAIS-III	123.72 (14.65)	122.18 (16.23)	*t*_84_ = −1.35	*p* = .182	BF_01_ = 2.01
Nonverbal IQ, RPM^a^	103.05 (11.07)	101.79 (10.29)	*t*_83_ = 0.54	*p* = .588	BF_01_ = 3.88
Social Anxiety, LSAS	51.42 (18.58)	15.23 (7.57)	*t*_55.56_ = 11.82	*p* < .001∗	BF_10_ > 100

Values are presented as *n* (%) for categorical variables or mean (SD) for continuous variables. BF_01_ indicates BF in favor of the null hypothesis of no group differences, and BF_10_ indicates BF in favor of the alternative hypothesis.

∗*p* Value < .05.

BF, Bayes factor; LSAS, Liebowitz Social Anxiety Scale; RPM, Raven’s Progressive Matrices; SA, social anxiety; WAIS-III, Wechsler Adult Intelligence Scale, Third Edition.

a Nonverbal IQ data were missing for 1 participant with low SA.

### Experimental Protocol

**Figure 1:**
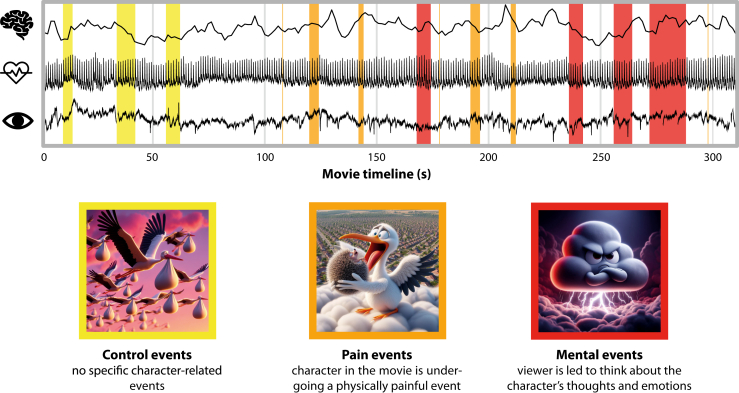
Experimental stimuli and multimodal recordings. Participants in the high and low social anxiety groups viewed a 6-minute animated film depicting the evolving friendship between a stork and a cloud while brain activity was continuously recorded in addition to heart rate and pupil size. The film, previously validated as a theory of mind localizer, featured 3 annotated event types: mental (eliciting inferences about characters’ thoughts and emotions), pain (depicting physical discomfort), and control (neutral scenes without foreground characters). Event images were artificial intelligence generated (Bing Copilot) to approximate original scenes.

Participants viewed the animated short *Partly Cloudy* (5 minutes 45 seconds), previously validated as a ToM localizer (38). The film portrays an evolving relationship between a stork and a cloud across episodes of cooperation, rejection, injury, and reconciliation. Following prior work, 3 event types were annotated (Figure 1): mental (44 seconds across 4 scenes), pain (26 seconds across 7 scenes), and control (24 seconds across 3 scenes). Mental scenes were selected for content likely to elicit inferences about characters’ mental states (e.g., distress, hope, betrayal). Pain scenes depicted physical discomfort (e.g., electric shocks, animal attacks), while controlling for affective salience in the absence of mental state inference. Control scenes consisted of visually engaging but socially neutral content (e.g., flying birds, empty skies). These annotations formed the basis for event-related analyses contrasting mental scenes with pain and control scenes to isolate mentalizing-related activity. The film was presented without interruption, enabling dynamic ISC analyses across the full duration of viewing.

To minimize state anxiety, participants were acclimated to the MRI environment using a mock scanner and instructed to simply watch the film while minimizing head motion. Following the scan, participants completed an unannounced written plot summary. Responses were scored by independent raters using a taxonomy distinguishing mental and emotional language from other content-related categories (53). Group differences in mental state references were assessed using independent sample *t* tests. A quasi-Poisson regression modeled the proportion of negatively valenced emotion words relative to each participant’s total emotion-related word count.

### Data Acquisition

Heart rate was recorded continuously at 5000 Hz using a finger pulse sensor connected to a BrainAmp ExG MR amplifier (BrainVision software). Pupil diameter was measured at 1000 Hz using an Eyelink 1000 Plus eye tracker. MRI data were acquired on a Siemens 3T scanner with a 32-channel head coil. High-resolution structural images were obtained using a T1-weighted magnetization-prepared rapid acquisition gradient-echo (MPRAGE) sequence (TR = 2200 ms, inversion time = 1100 ms, TE = 2.6 ms, flip angle = 11°, voxel size = 0.8 mm isotropic, acceleration factor = 2). Functional images were acquired using a multiband multiecho sequence (TR = 1500 ms, TEs = 13.4/34.8/56.2 ms, flip angle = 75°, voxel size = 2.5 mm isotropic, acceleration factor = 2). Head motion did not differ between groups, as assessed by mean framewise displacement (mean_high-SA_ [SD] = 0.16 [0.08], mean_low__-__SA_ = 0.16 [0.07], *t*_84_ = 0.20, *p* = .841, Bayes factor [BF]_01_ = 4.36), maximum displacement (mean_high__-__SA_ = 0.97 [1.27], mean_low__-__SA_ = 1.15 [1.13], *t*_84_ = 0.67, *p* = .507, BF_01_ = 3.65), and total translational (mean_high__-__SA_ = 118.48 [110.45], mean_low__-__SA_ = 107.42 [78.70], *t*_84_ = −0.53, *p* = .594, BF_01_ = 3.92) and rotational (mean_high__-__SA_ = 2.29 [2.05], mean_low__-__SA_ = 2.37 [1.91], *t*_84_ = 0.18, *p* = .856, BF_01_ = 4.38) movement.

### Heart Rate Analysis

Scanner artifacts were removed using a deconvolution filter (BrainAmpConverter toolbox), followed by bandpass filtering between 0.2 Hz and 3 Hz to isolate physiologically relevant frequencies (54). Heartbeats were detected using a 600-ms peak detection window, and interbeat intervals were used to derive continuous heart rate time series. Autonomic arousal was assessed by comparing mean heart rate trajectories across the film and by quantifying movie-driven alignment using dynamic ISC. For each participant, ISC indexed the similarity between their time series and the leave-one-out average of the corresponding group (55), computed using a 30-second sliding window with 100-ms steps. Group differences were evaluated using cluster-based permutation testing applied to independent samples *t* tests, with 10,000 Monte Carlo permutations and a significance threshold of *p* < .05 (56).

### Pupillometry Analysis

Blink- and saccade-related artifacts were removed using noise-based detection and adaptive velocity thresholding (57,58), with additional outliers excluded by visual inspection (59). Pupil time series were *z* scored and corrected for luminance fluctuations using fifth-order polynomial regression in R (60), with framewise brightness values computed according to Rec. 709 standards (61). Event-related pupil responses were analyzed using a 3 (event type: mental, pain, control) × 2 (group: high SA, low SA) mixed-design analysis of variance (ANOVA) on mean pupil diameter. Significant interactions were followed by independent samples *t* tests with a Bonferroni-corrected significance threshold of *p* < .017 (.05/3 tests). Dynamic ISC of pupil diameter was computed using the same leave-one-out sliding-window approach as for heart rate (see Heart Rate Analysis above).

### fMRI Analysis

Preprocessing was performed in SPM12. Multiecho images were combined into a single time series, realigned using second-degree B-spline interpolation, and unwarped using field maps. Functional images were then coregistered to structural images, normalized to Montreal Neurological Institute (MNI) space, and spatially smoothed with an 8-mm full width at half maximum Gaussian kernel. First-level general linear models included regressors for mental, pain, and control events, as well as the film’s end credits. Motion parameters (including derivatives, squared terms, and cubic terms) and signals from white matter, cerebrospinal fluid, and nonbrain voxels were included as nuisance regressors.

To assess ToM engagement, 2 mixed-design ANOVAs tested effects of event type (mental > pain; mental > control) and group. A conjunction-null analysis identified regions consistently engaged across both contrasts. Statistical significance was assessed using cluster-level familywise error (FWE) correction (*p*_FWE_ < .05, voxelwise *p* < .001), and anatomical labels were assigned using the SPM Anatomy Toolbox (62). Bayesian analyses quantified evidence for the absence of group differences in canonical ToM regions by extracting activation estimates from 8-mm spheres centered on conjunction peaks and computing BFs (63). To control for temporal structure, an additional first-level regressor indexing linear progression through the film was included; this did not alter the results, indicating robustness to event timing.

Movie-driven neural alignment was assessed using voxelwise dynamic ISC applied to motion- and noise-corrected fMRI time series (42). ISC was computed using the same leave-one-out sliding-window approach, applied to downsampled data for computational efficiency (7.5-mm spatial and 4.5-second temporal resolution). Group differences were identified using cluster-based permutation testing. For visualization, *t* values were summed across time points to generate 3-dimensional statistical maps, and spatial overlap with ToM activation was quantified as the proportion of overlapping voxels within each significant cluster.

## Results

### Postviewing Movie Assessment

**Figure 2:**
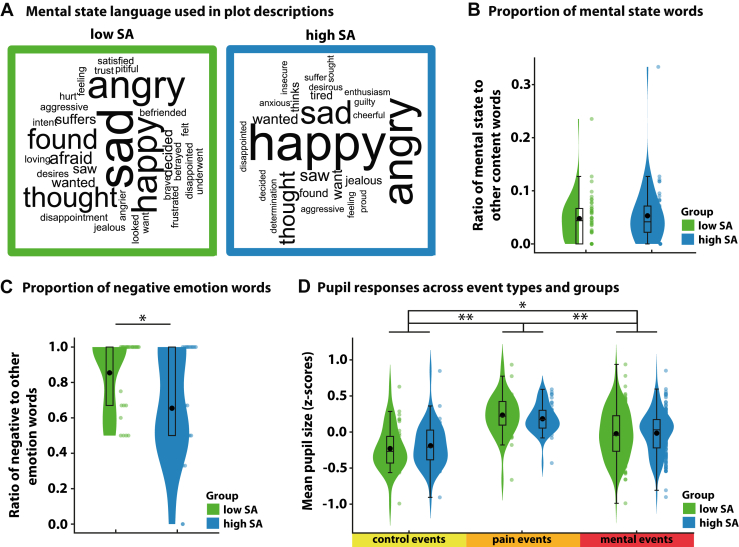
Language use and pupil responses. **(A)** Word clouds depicting the relative frequency of mental state terms in postviewing summaries for the high and low social anxiety (SA) groups. **(B)** The proportion of mental state terms did not differ between groups. **(C)** Participants in the high-SA group used a lower proportion of negatively valenced emotion words. **(D)** Pupil diameter differed by event type, with no group effects. Violin plots show full distributions; boxplots indicate means (black dots), medians, and IQRs. ∗*p* < .05, ∗∗*p* < .001.

Following the film, participants summarized the plot in their own words. The high- and low-SA groups did not differ in the proportion of mental state terms used (mean_high__-__SA_ = 0.053 [0.057], mean_low__-__SA_ = 0.048 [0.064], *t*_82_ = 0.47, *p* = .642, BF_01_ = 4.00) (Figure 2A, B) or in the overall use of emotion-related language (mean_high__-__SA_ = 0.042 [0.057], mean_low__-__SA_ = 0.034 [0.038], *t*_82_ = 0.73, *p* = .469, BF_01_ = 3.49). Contrary to predictions, however, individuals in the high-SA group used a lower proportion of negatively valenced emotion words in their summaries (mean_high__-__SA_ = 0.65 [0.31], mean_low__-__SA_ = 0.85 [0.21], *t*_50_ = −2.61, *p* = .012) (Figure 2C), indicating a more positive emotional framing of the narrative.

### Heart Rate Responses

Heart rate fluctuated over the course of the film but followed highly similar trajectories across groups. No group differences were observed in mean heart rate (mean_high__-__SA_ = 65.45 [8.69], mean_low__-__SA_ = 64.11 [9.90], *t*_72_ = 0.62, *p* = .536, BF_01_ = 3.52) (Figure S1A) or in movie-driven alignment of heart rate dynamics as indexed by dynamic and mean ISCs (mean_high__-__SA_ = 0.22 [0.13], mean_low__-__SA_ = 0.21 [0.10], *t*_72_ = 0.23, *p* = .81, BF_01_ = 4.06) (Figure S1B). These results indicate comparable autonomic arousal across groups throughout the movie.

### Pupil Responses

Pupil diameter varied robustly as a function of event type but showed highly similar response profiles across groups. Dilation was greatest during pain events, followed by mental and control events (*F*_2,146_ = 33.93, *p* < .001, BF_10_ > 100; pain > mental: *t*_74_ = 2.72 *p* = .008, *d* = 0.31; mental > control: *t*_74_ = 7.91, *p* < .001, *d* = 0.91) (Figure 2D). No main effects or interactions involving group were observed (group: *F*_1,73_ = 0.10, *p* = .750, BF_01_ = 6.22; group × event: *F*_2,146_ = 0.48, *p* = .619, BF_01_ = 6.66). Dynamic ISC analyses revealed pronounced fluctuations in pupil alignment across viewers but no group differences at any time point or in mean ISC (mean_high__-__SA_ = 0.58 [0.24], mean_low__-__SA_ = 0.61 [0.19], *t*_73_ = 1.09, *p* = .280, BF_01_ = 2.51) (Figure S2). Together, these findings indicate comparable event-related and continuous attentional engagement across groups.

### ToM Engagement

**Figure 3:**
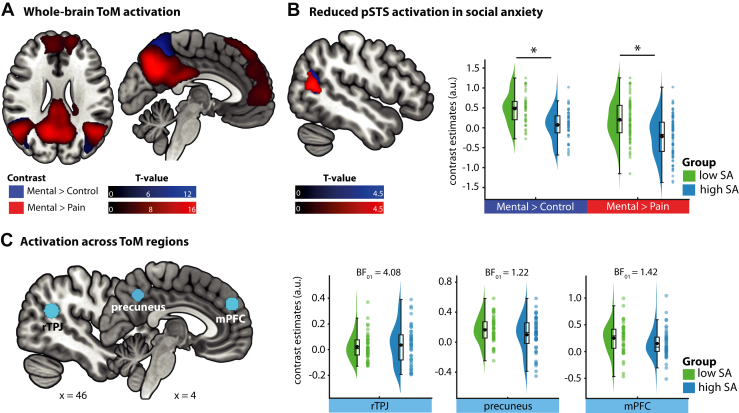
Theory of mind (ToM) engagement. **(A)** Canonical ToM network engaged during mental scenes (mental > control in blue; mental > pain in red). **(B)** Reduced activation in the left posterior superior temporal sulcus (pSTS) in participants with high social anxiety (SA) across both contrasts. **(C)** No group differences in other ToM regions, with Bayes factors supporting the null hypothesis (BF_01_). Contrast estimates reflect mean activation averaged across contrasts. ∗*p* < .05. a.u., arbitrary unit; mPFC, medial prefrontal cortex; rTPJ, right temporoparietal junction.

Whole-brain contrasts revealed robust engagement of the canonical ToM network during mental events relative to both pain and control scenes (Figure 3A and Table S1). Across participants, this network included the bilateral TPJ (MNI [x, y, z]: right TPJ [rTPJ] [46, −52, 22]; left TPJ [−44, −60, 22]), precuneus ([0, −54, 42]), and mPFC ([6, 50, 32]), all significant at *p*_FWE_ < .001. Within this otherwise preserved network, participants in the high-SA group showed selectively reduced activation in the left posterior STS (pSTS) across both mentalizing contrasts (mental > control: [−44, −62, 18], *t* = 4.41, *p*_FWE_ = .003, BF_10_ > 100; mental > pain: [−50, 64, 14], *t* = 4.58, *p*_FWE_ = .009, BF_10_ > 100) (Figure 3B). Bayesian analyses provided evidence against group differences in other core ToM regions (rTPJ: BF_01_ = 4.08; precuneus: BF_01_ = 1.22; mPFC: BF_01_ = 1.42), indicating largely preserved ToM engagement outside this focal region.

### Movie-Driven Neural Alignment

**Figure 4:**
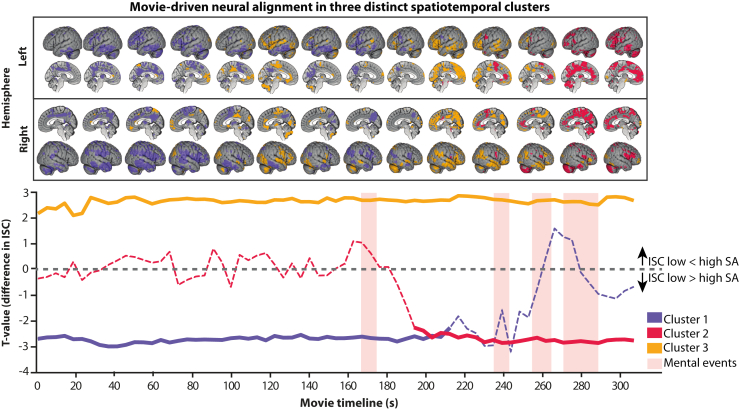
Movie-driven neural alignment. Dynamic intersubject correlation (ISC) identified 3 spatiotemporal clusters with group differences in neural alignment. Participants with high SA showed reduced alignment in clusters 1 and 2 and increased alignment in cluster 3. Solid lines indicate time intervals with group differences. Pink bands indicate mental events.

**Figure 5:**
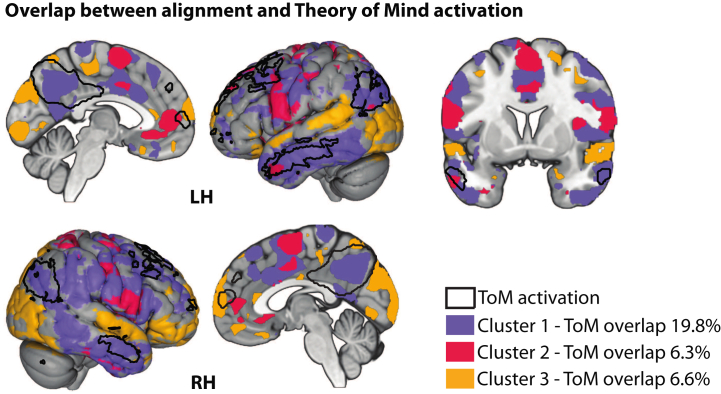
Limited overlap between alignment and theory of mind (ToM) activation. Intersubject correlation clusters showed minimal spatial overlap with ToM regions (outlined), with no cluster exceeding 20%. For visualization purposes, clusters are displayed at a cumulative *t* value threshold of ≥20. LH, left hemisphere; RH, right hemisphere.

Dynamic ISC analyses identified 3 spatiotemporal clusters showing group differences in neural alignment during movie viewing (Figure 4). Two clusters showed reduced alignment in participants in the high-SA group. Cluster 1 spanned the first two-thirds of the film (cluster stat = 8585, *p* = .003) and included superior parietal ([24, −52, 74], *t*_max._ = 5.54, *t*_sum_ = 105.26) and bilateral temporal (left: [−60, −10, −22], *t*_max._ = 4.06, *t*_sum_ = 94.37; right: [66, −40, −10], *t*_max._ = 5.04, *t*_sum_ = 95.64) regions. Cluster 2 emerged during the final one-third of the film (cluster stat = 4497, *p* = .02) and encompassed supplementary motor ([0, 8, 62], *t*_max._ = 4.39, *t*_sum_ = 56.54), medial prefrontal ([−6, 50, 8], *t*_max._ = 6.77, *t*_sum_ = 56.01), and precentral ([−54, 2, 20], *t*_max._ = 3.84, *t*_sum_ = 51.21) regions. In contrast, cluster 3 showed increased alignment in participants in the high-SA group across much of the film (cluster stat = −8478, *p* = .002), involving occipital ([42, −70, 8], *t*_min_ = −3.96, *t*_sum_ = −110.16), inferior frontal ([30, 32, −10], *t*_min_ = −4.91, *t*_sum_ = −108.48), and superior temporal ([48, 2, −10], *t*_min_ = −3.81, *t*_sum_ = −97.43) cortices. Spatial overlap between ISC clusters and the ToM network was limited, with no cluster exceeding 20% overlap (Figure 5). Post hoc analyses revealed no association between ISC values within these clusters and individual pSTS activation among participants in the high-SA group (all *p*s > .20), indicating that alignment effects were not directly coupled to ToM-related activation.

### Comparison With Autism

**Figure 6:**
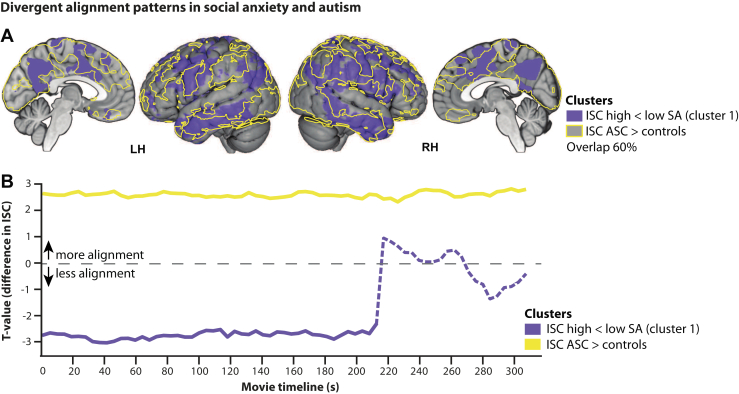
Divergent alignment patterns in social anxiety (SA) and autism. **(A)** Cluster 1 overlapped substantially with a previously reported autism-related cluster. **(B)** Despite anatomical overlap, alignment was reduced in high-SA participants but increased in individuals with autism, relative to the control participants. ASC, autism spectrum condition; ISC, intersubject correlation; LH, left hemisphere; RH, right hemisphere.

Because cluster 1 closely overlapped with a cluster previously reported in individuals with autism spectrum condition (ASC) using the same paradigm (42), we conducted an exploratory post hoc cross-study comparison. The ASC and high-SA groups were demographically matched and comparable in SA but differed in autism traits (Table S2). Spatial overlap between clusters was substantial (∼60%), spanning supramarginal, inferior temporal, anterior midcingulate, and precuneus regions (Figure 6A). Despite this anatomical convergence, alignment patterns diverged: Participants with high SA showed reduced alignment, whereas individuals with ASC showed increased alignment across much of the film (Figure 6B). These findings suggest distinct modes of social information processing, characterized by more idiosyncratic responses in SA and more stereotyped responses in autism relative to controls.

## Discussion

By leveraging a movie paradigm that reliably engages the ToM network during well-defined scenes, this study provides converging neural evidence relevant to two sociocognitive accounts of SA: altered mentalizing and broader interpretive biases. Individuals in the high-SA group showed reduced activation in the left pSTS, a core subregion of the ToM network, despite otherwise preserved engagement of canonical mentalizing regions. These focal ToM-related differences co-occurred with more widespread alterations in movie-driven neural alignment outside the ToM network, revealed by ISC analyses and observed in the absence of group differences in physiological arousal or attentional engagement. Together, these findings support an integrative neurocognitive account in which SA is characterized by both circumscribed alterations in ToM-related computations and broader shifts in the neural organization and interpretation of social information, even under minimally evaluative conditions.

Our results extend prior behavioral evidence of reduced performance on emotion recognition and mental state attribution tasks in SA (12) by identifying corresponding neural differences during implicit movie viewing. Participants in the low-SA group showed selective recruitment of the left pSTS during scenes requiring inferences about characters’ thoughts and emotions, whereas individuals in the high-SA group exhibited attenuated scene-specific modulation despite preserved engagement of the broader ToM network. This pattern points to a focal alteration in mentalizing rather than a global impairment and demonstrates that ToM-related differences in SA can emerge even in the absence of explicit task demands or evaluative pressure (10).

The pSTS plays a central role in integrating perceptual social cues with internal models of others’ intentions (64,65). Through its connectivity with motion- and face-sensitive regions, including the middle temporal area and the fusiform face area, it supports the interpretation of dynamic social signals such as gaze, biological motion, and facial expressions (66, 67, 68, 69, 70, 71, 72). The left pSTS, in particular, has been implicated in decoding others’ emotional states, with variation in its structure and function being associated with differences in social perception across contexts, including loneliness and SA (73, 74, 75, 76). Therefore, reduced engagement of this region during movie viewing may reflect differences in how socially salient affective information is integrated during ongoing perception (77), consistent with group differences in emotional framing observed in postviewing descriptions.

Beyond these focal ToM effects, dynamic ISC analyses revealed region-specific differences in neural alignment indicative of broader alterations in social information processing in SA. Increased alignment in early sensory regions, including the occipital and superior temporal cortex, suggests more stimulus-locked and uniform perceptual processing. In contrast, reduced alignment in frontoparietal and temporal association cortices indicates greater divergence at higher levels of processing, consistent with more individualized, internally guided interpretations of social content (35,36,78,79). Framed within hierarchical models of cortical organization (80), this pattern points to a redistribution of social information processing across the cortical hierarchy, with increasingly idiosyncratic integration at higher-order association levels. Crucially, these effects occurred despite comparable heart rate and pupil dynamics across groups, inconsistent with differences in arousal or overall attentional engagement as their primary source. Instead, the observed redistribution may reflect an integrative bias that, in contexts involving expectations of negative evaluation, could influence how social information is weighted toward threat-relevant interpretations.

An additional insight emerged from the substantial spatial overlap between one alignment cluster identified here and a network previously reported in autistic individuals using the same paradigm (42). Despite this anatomical convergence, alignment effects diverged in direction; i.e., they were reduced in SA and increased in autism. This dissociation indicates that similar neural systems can support qualitatively distinct modes of social information processing across conditions. Whereas increased alignment in autism may reflect more stereotyped or stimulus-driven processing, reduced alignment in SA may index more idiosyncratic, internally guided interpretations. Notably, the 2 groups were comparable in SA severity, suggesting that similar behavioral phenotypes can arise from distinct neural mechanisms. Whereas SA in autism may emerge secondarily from chronic social difficulties (48), SA disorder may be more closely linked to early-emerging interpretive tendencies that prioritize evaluative appraisal over perceptual evidence (81,82). Together, these findings highlight movie-driven neural alignment as a transdiagnostic marker of how social information is processed rather than a simple index of anxiety severity.

Contrary to predictions, participants in the high-SA group used fewer negatively valenced emotion words in their postviewing summaries. This effect was not accompanied by differences in overall mental state or emotion language, suggesting a selective shift in emotional framing rather than reduced engagement with the narrative. Several explanations are possible. Because the assessment was offline and explicit, individuals in the high-SA group might have engaged in impression management or self-monitoring, downplaying negative content (83,84). Alternatively, the film’s positive resolution might have disproportionately shaped retrospective evaluations. It is also possible that negativity biases in SA are more pronounced in self-referential or interactive contexts than in evaluations of fictional social content (33,47,85,86). Future studies incorporating real-time affective or experiential measures could help adjudicate between these possibilities.

We found no direct relationship between pSTS activation during mentalizing events and broader patterns of movie-driven neural alignment, suggesting that these measures index partially independent dimensions of sociocognitive processing. Although some theoretical models posit a tradeoff between internal monitoring and mentalizing resources (25,26), our data do not support a simple antagonistic relationship. Instead, this dissociation is consistent with an integrative neurocognitive account in which SA reflects multiple, not obligatorily coupled, vulnerabilities. In this framework, elevated SA may arise from biased interpretive processing in some individuals, altered weighting of mental state inferences in others, or the co-occurrence of both in the same individual (10). These neurocognitive contributions may operate at different levels of the processing hierarchy and be differentially expressed depending on contextual and evaluative demands.

Several limitations of the current study warrant consideration. First, although the movie-viewing paradigm enabled the study of implicit mentalizing during continuous social perception, emotional and mental state content were partially correlated. While the pain condition controlled for affective salience, it did not eliminate emotional mental state processing, such that the mental condition did not isolate emotion-free mental state inference. Second, behavioral assessment was limited to an unannounced postviewing summary, capturing retrospective interpretation rather than online processing. As a result, the current data cannot directly link moment-to-moment neural dynamics to unfolding interpretive processes during the narrative (87). Finally, participants were grouped using validated self-report measures indexing continuous variation in SA rather than categorical diagnoses, and comorbid conditions were not systematically assessed. While this dimensional approach enhances sensitivity to individual differences in symptom severity (5,88), it limits the conclusions that can be drawn about diagnostic specificity and clinical boundaries.

### Conclusions

This study identifies converging neurocognitive signatures of SA during implicit movie viewing, linking focal alterations in ToM-related processing with broader shifts in movie-driven neural alignment across the cortical hierarchy. Reduced alignment in higher-order association regions, observed independently of arousal or attentional differences, suggests more individualized modes of social information processing in SA, while opposite patterns in autism underscore the transdiagnostic potential of movie-driven neural alignment.

## Supplementary Material

Figures S1–S2 and Tables S1–S2

Key Resources Table
