# Supplementary material for: Altered Theory of Mind Engagement and Neural Alignment in Social Anxiety During Movie Viewing
Source: Biol Psychiatry Glob Open Sci. 2026 Mar 14;6(4):100721. doi: 10.1016/j.bpsgos.2026.100721 (PMC13126348; doi:10.1016/j.bpsgos.2026.100721)
Supplement: Figures S1–S2 and Tables S1–S2 [file mmc1.pdf]

SUPPLEMENTARY INFORMATION

Altered Theory of Mind Engagement and Neural Alignment in Social Anxiety During Movie Viewing

Koch *et al.*

Supplemental information

Table S1. Within-subjects fMRI results for two contrasts assessing ToM activation.

Cotrast	*Anatomical location*	*Cluster size*	*MNI coordinates*			*T-value*	p_{FWE} *cluster*
			x	*y*	*z*		
**Mental > Control**	Precuneus extending into TPJ	17625	2	-54	46	13.41	< .001
	Right temporoparietal junction	-	52	-54	20	10.71	-
	Left temporoparietal junction	-	-46	-60	24	9.37	-
	Left fusiform gyrus	2207	-28	-48	-8	10.07	< .001
	Right middle temporal gyrus	1897	56	2	-26	9.57	< .001
	Left middle temporal gyrus	1862	-52	4	-28	8.81	< .001
	Left middle frontal gyrus	5636	-32	10	48	6.37	< .001
	Medial prefrontal cortex	-	6	54	26	6.34	-
	Right postcentral gyrus	396	42	-22	38	5.40	.006
	Left middle orbital gyrus	240	2	62	-10	4.80	.044
	Right thalamus	305	18	-26	10	4.68	.018
**Mental > Pain**	Precuneus extending into TPJ	36624	6	-64	42	16.68	<.001
	Right temporoparietal junction	-	44	-58	30	13.81	-
	Left temporoparietal junction	-	-44	-60	28	14.49	-
	Left middle temporal gyrus	2211	-54	0	-32	10.74	<.001
	Right middle temporal gyrus	1425	60	-10	-20	8.36	<.001
	Right parahippocampal gyrus	304	20	-36	-12	5.94	.023
**Conjunction**	Precuneus	5373	0	-54	42	12.87	< .001
	Right temporoparietal junction	1956	46	-52	22	10.35	< .001
	Left temporoparietal junction	1957	-46	-60	22	10.12	< .001
	Left middle temporal gyrus	1603	-52	4	-28	8.87	< .001
	Right middle temporal gyrus	1251	58	-12	-18	8.18	< .001
	Left middle frontal gyrus	4740	-32	8	46	6.53	< .001
	Medial prefrontal cortex	-	6	50	32	6.26	-

Table S2. Demographic comparison of the Autism Spectrum Condition (ASC) and High SA groups.

	Group		***Group difference***		
	High SA (n = 43)	*ASC (n = 52)*	*Test statistics*		*Bayes Factor*
Gender (female)	27 (62.79%)	29 (55.77%)	$\chi^2_{(1)} = 0.23$	$p = .629$	$BF_{01} = 3.19$
Age (years)	26.33 (5.92)	27.71 (6.41)	$t_{(93)} = -1.09$	$p = .280$	$BF_{01} = 2.75$
Verbal IQ (WAIS-III)	123.72 (14.65)	125.80 (15.88)	$t_{(92)} = -0.66$	$p = .513$	$BF_{01} = 3.80$
Nonverbal IQ (RPM)	103.05 (11.07)	102.59 (9.0-)	$t_{(92)} = 0.21$	$p = .833$	$BF_{01} = 3.88$
Autism traits (AQ-50)	19.16 (7.67)	30.61 (9.04)	$t_{(92)} = -6.55$	$p < .001$	$BF_{10} > 100$
Social anxiety (LSAS)	51.42 (18.58)	52.37 (24.84)	$t_{(92)} = -0.21$	$p = .836$	$BF_{01} = 4.51$

Values are presented as frequency (%) for categorical variables or mean (SD) for continuous variables. SA, Social Anxiety; WAIS, Wechsler Adult Intelligence Scale; RPM, Raven's Progressive Matrices; AQ-50, Autism-Spectrum Quotient; LSAS, Liebowitz Social Anxiety Scale; BF_{01}, Bayes Factor in favor of the null hypothesis of no group differences; BF_{10}, Bayes factor in favor of the alternative hypothesis of group differences.

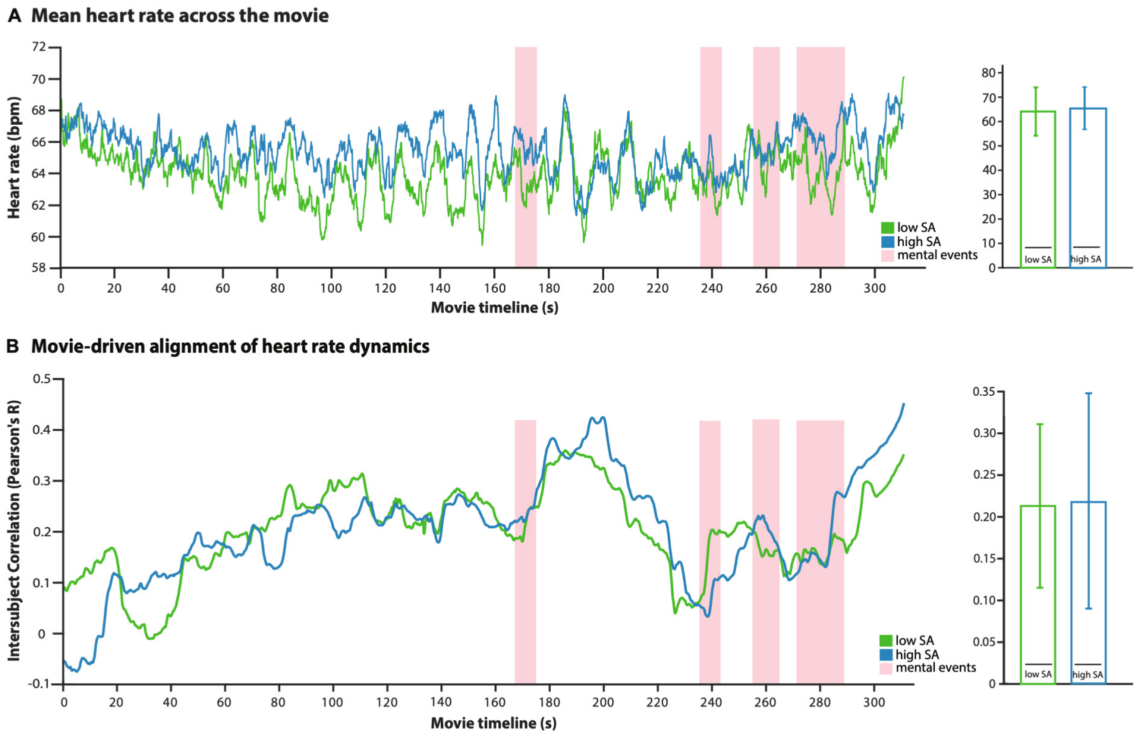

Figure S1. Heart rate dynamics during movie viewing. (A) Mean heart rate time course across the film for socially anxious and low-anxiety participants. Adjacent bar graphs show average heart rate across the full movie duration (± SD). **(B)** Dynamic ISC of heart rate responses across viewers. No group differences were observed in mean heart rate or heart rate alignment. bpm, beats per minute; SA, social anxiety

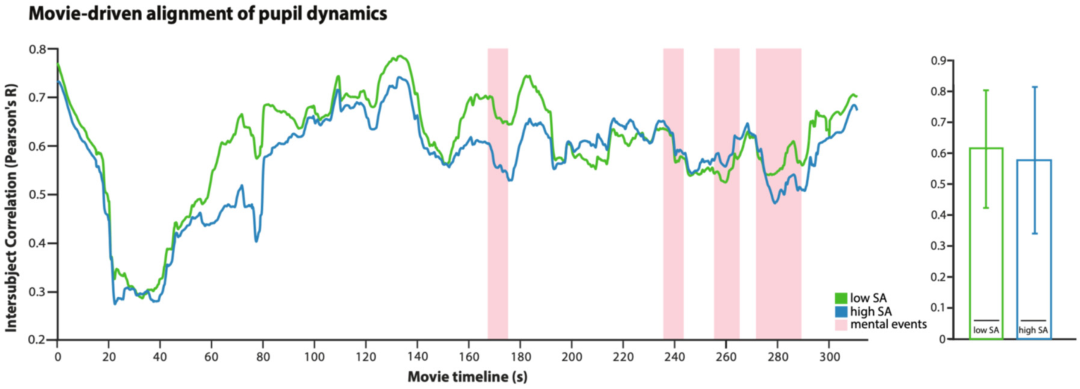

Figure S2. Movie-driven alignment of pupil dynamics. Dynamic ISC revealed pronounced fluctuations in pupil response alignment across the film. No significant group differences were observed at any time point.
